# Remote Online-Based Application of the Cerebellar Cognitive Affective Syndrome Scale

**DOI:** 10.1007/s12311-026-02097-w

**Published:** 2026-10-07

**Authors:** Sönke Konrad, Johanna Aschmann, Raquel van der Veen, Kerstin Rubarth, Frank Konietschke, Dagmar Timmann, Andreas Thieme

**Affiliations:** 1 Department of Neurology, Essen University Hospital, University of Duisburg-Essen, Hufelandstraße 55, Essen, 45147 Germany; 2 Center for Translational Neuro- and Behavioral Sciences (C-TNBS), Essen University Hospital, University of Duisburg-Essen, Essen, Germany; 3 Corporate Member of Freie, Institute for Biometry and Clinical Epidemiology, Charité – Universitätsmedizin Berlin, Universität Berlin and Humboldt Universität zu Berlin, Charitéplatz 1, Berlin, 10117 Germany; 4 Berlin Institute of Health (BIH), Berlin, Germany; 5 Department of Neurology, Knappschaft-Kliniken Bottrop, Academic Teaching Hospital of the University of Duisburg-Essen, Bottrop, Germany

**Keywords:** Cerebellar cognitive affective syndrome, CCAS-S, Remote assessment, Telemedicine, Cerebellar ataxia, Neuropsychological testing

## Abstract

The Cerebellar Cognitive Affective Syndrome Scale (CCAS-S) is increasingly used in clinical trials to assess non-motor function in cerebellar disorders, yet its suitability for remote administration has not been evaluated. Using a matched-pairs design, we compared remote, video-based administration of the German CCAS-S (version A) in 22 patients with cerebellar disease and 22 matched controls with at site -testing from a larger, previously tested cohort. Group matching included age, level of education, sex, occupational experience, and ataxia severity. Uncorrected as well as age, education and sex corrected CCAS-S scores were analysed. Test-retest and interrater reliability were assessed in subsets of the remote cohort. Remotely tested patients did not perform worse than at site tested patients regarding the number of failed test items or the total sum raw score. Instead, fewer significant group differences on single test items emerged in the remote cohort between patients and controls compared to the at site tested cohort. Test-retest reliability of remote CCAS-S administration was good in patients and in controls, and interrater reliability was good to excellent. The coefficients were of a similar magnitude as the published values for at site administration, but were not compared statistically because of differences in study design and small subgroup sizes. Remote administration of the CCAS-S is feasible and shows good test-retest and interrater reliability. However, the present cohort-comparison design does not permit conclusions regarding measurement equivalence between remote and at site administration. Until equivalence has been established in a within-subject crossover design, remote and at site CCAS-S data should not be pooled, and remotely assessed patients should be compared against remotely assessed controls.

**Supplementary Information:**

The online version contains supplementary material available at https://doi.org/10.1007/s12311-026-02097-w.

## Introduction

It is now widely accepted that the cerebellum is involved in non-motor functions [1–4] besides its long know role in motor coordination and balance control [2, 5, 6]. Consequently, disorders of the cerebellum (i.e. ataxias) result in motor and non-motor symptoms [7]. Among the most disabling motor manifestations is reduced mobility, particularly due to gait and postural instability, which is closely associated with overall disease severity [8, 9] and leads to reduced social participation [10–12]. The non-motor symptoms are also known to impair quality of life [12, 13].

The non-motor symptoms in ataxias are referred to as *Cerebellar Cognitive Affective Syndrome (CCAS)* and usually involve problems with executive, linguistic and visuospatial cognitive functions as well as neuropsychiatric symptoms [14]. The neuropsychiatric symptoms observed in CCAS are affective, psychosis- or autism-like symptoms and problems with social skill [13–16].

Ataxias comprise a heterogeneous group of rare neurodegenerative movement disorders. Until recently, effective disease-modifying treatments for ataxia were largely unavailable [17]. The approval of omaveloxolone as the first Food and Drug Administration (FDA) and European Medicines Agency (EMA) -approved targeted therapy for Friedreich’s ataxia (FRDA) has therefore marked an important turning point [18]. In parallel, targeted therapies for other hereditary ataxias, including the most common forms such as spinocerebellar ataxia (SCA) types 1, 2, 3 and 6, are currently under development or being evaluated in early-stage clinical trials (see https://www.ataxia.org/pipeline/ for an up-to-date overview).

With the prospect of increasing clinical trial activity, there is a growing need for outcome measures that are both sensitive to change and feasible for repeated use [19]. This need is particularly pressing given the mobility limitations of many patients with ataxia, which can make frequent at site assessments burdensome. Consequently, assessment tools that can be administered remotely in the patient’s home are of considerable interest. Recently, a remotely administered version of the *Scale for the Assessment and Rating of Ataxia* (SARA) has been developed and validated [20, 21]. One key advantage of remote assessments is the ability to capture day-to-day fluctuations through repeated home-based testing, which may provide a more ecologically valid representation of the disease’s impact on daily life.

While remote administration has been established for ataxia (i.e. motor) assessments [20, 21], the feasibility of home-based cognitive testing in ataxia patients remains more unclear. Although, some studies have applied the Montreal Cognitive Assessment [22, 23], a screening test for dementia, remotely to patients with ataxia, remote testing of the Cerebellar Cognitive Affective Syndrome Scale (CCAS-S), which is specifically designed to assess the non-motor manifestations in ataxias [24], has not yet been validated in this patient population. Two recent studies have nevertheless applied the CCAS-S outside the classical at site setting. Selvadurai et al. administered the scale entirely online via teleconferencing in individuals with spinocerebellar ataxia types 1, 2, 3 and 6 and in demographically matched controls, and explicitly noted that their online results may not be directly comparable to previous at site assessments [25]. Bolzan et al. assessed manifest and premanifest carriers of spinocerebellar ataxia type 3/Machado-Joseph disease as well as controls either at site or in virtual settings and reported cognitive-affective deficits, captured most sensitively by the CCAS-S, already before the onset of ataxia [26]. Because of the COVID-19 pandemic, they changed the mode of assessment to remote. They added the mode of assessment as a covariate in their models and observed better CCAS-S performance in all remotely assessed subgroups than in those assessed at site. A formal comparison between at site and remote administration would have been desirable, but was not feasible within their study design because no participants were tested at site as well as remote, and participants were not matched.

The CCAS-S is a brief bedside screening tool which has initially been published 2018 in American English [24]. It has since been translated and adopted into other languages [27–32] including German [33], facilitating more standardized and time-effective cognitive screening of cerebellar patients. Importantly, the CCAS-S enables multinational, multicenter studies with larger cohorts and allows for more reliable comparisons of cognitive outcomes across sites and studies [34]. Besides these advantages, the CCAS-S has also some limitations that have to be kept in mind when administering the scale. These include the effect of demographic variables [28, 35–37] and possibly dysarthria [32, 38] on test results, a high rate of false positive test results [28, 32, 35, 36, 39], insufficient sensitivity in mildly cognitively impaired patients [39] and high values for minimal detectable change in follow-up examinations [35, 40]. However, in lack of better validated cognitive tests to assess the CCAS-S, it is recommended by ataxia experts for upcoming clinical trials [19].

A study designed to compare remote with at site administration of the CCAS-S in matched cohorts has, to our knowledge, not yet been reported. In the present study, using a matched-pairs design, we compared a remote application of the German version A of the CCAS-S in patients with various cerebellar disorders and healthy controls with the standard at site testing conducted at the ataxia outpatient clinic of the University Hospital Essen.

## Methods

### Study Population

In total, 27 patients with cerebellar disease and 26 healthy controls, matched for age (± 5 years, yrs), educational level (± 3 yrs of education), occupational experience (± 15 yrs of occupation), and sex, were tested remotely via video conference using the Zoom application (versions 5.4 and 5.5; https://www.zoom.com) after providing written informed consent. The original validation study of the German CCAS-S [33, 35] was initiated prior to the COVID-19 pandemic, and remote testing was therefore not part of the preregistered study protocol mentioned below. During the pandemic, a subset of patients was assessed remotely via video conference as an alternative to at site visits. The data of remote testing were not included in the original validation study, but were retained for the present comparative analysis. None of the participants tested remotely were part of the cohort used in the German CCAS-S validation study [33, 35]. Five remotely tested patients were excluded because they met exclusion criteria, two were excluded for medical reasons (one because of psychiatric disorder and one for regular increased alcohol consumption), three were excluded due to incomplete data. In addition, 22 patients and 22 controls tested at site at the participating study sites were selected from the cohort of the German validation study of version A of the CCAS-S. Patients and controls tested at site were matched to those tested remotely with respect to age, level of education, occupational experience, and sex category according to the above mentioned criteria. Moreover, patients tested remotely were matched to patients tested at site regarding ataxia severity, as measured by the *Scale for the Assessment and Rating of Ataxia (SARA)* [8] and the *International Cooperative Ataxia Rating Scale (ICARS)* [41], the degree of extracerebellar involvement measured by the *Inventory of Non-Ataxia Signs (INAS)* [42], disease duration (in yrs), and classification as either *cerebellar-pure disease (cer-pure)* or *cerebellar-plus disease (cer-plus)*, as described below:

*Cerebellar-pure disease (cer-pure)* refers to disorders in which pathology is confined primarily to the cerebellum, such as spinocerebellar ataxia type 6 (SCA6) or cerebellar stroke without brainstem involvement. In contrast, *cerebellar-plus disease (cer-plus)* includes conditions with additional extracerebellar involvement, such as degenerative ataxias known to affect other brain regions (e.g., SCA1, SCA2, or SCA3) or cerebellar lesions extending to other structures, like the brainstem (for details: see Thieme et al. 2020 [33]).

Note that both the SARA and ICARS were included to allow cross-referencing with prior studies reporting either measure. For remotely tested patients, SARA and ICARS scores were obtained from documentation of concurrent at site clinic visits, conducted separately from and independently of the remote CCAS-S assessment.

Based on medical history and neurological examination, none of the participants included in the final dataset had psychiatric or neurological disorders other than isolated cerebellar stroke (patient characteristics are shown in Table 1). All participants were native German speakers. Participants were recruited at the University Hospital Essen.

**Table 1:** Demographic and clinical characteristics of patients and controls

	Male/ Female	Age (yrs)	Yrs of education	Yrs of occupation	Cer-pure/ Cer-plus Disease	Yrs diseased	ICARS - Score	SARA-Score	INAS- Count
patients tested remotely	8/14	54 ± 10.3	14 ± 2.7	27 ± 13.2	9/13	14.3 ± 14.1	33.4 ± 17.7	11.8 ± 6.8	3.0 ± 2.2
controls tested remotely	10/12	54 ± 14.6	16 ± 2.9	25 ± 15.3	-				
patients tested at site	8/14	55 ± 13.0	14 ± 2.7	27 ± 14.3	9/13	15.6 ± 13.5	37.2 ± 15.4	14.1 ± 6.2	3.3 ± 1.6
controls tested at site	8/14	53 ± 11.0	15 ± 3.7	21 ± 11.6	-				

The table shows the absolute numbers of patients or means ± standard deviations of demographic and clinical variables for each group

Abbreviations: *yrs*  years, *Cer-pure Disease*  diseases that primarily affect the cerebellum (for details see methods section), *Cer-plus Disease * diseases that affect extracerebellar structures in addition to the cerebellum (for details see methods section), *SARA* Scale for the Assessment and Rating of Ataxia, *ICARS*  International Cooperative Ataxia Rating Scale, *INAS*  Inventory of Non-Ataxia Signs

The study was approved by the local ethics committee (ethics no. 18-8444-BO) and was conducted in accordance with the principles of the current version of the Declaration of Helsinki. The study was prospectively registered in the German Clinical Study Register (DRKS; DRKS-ID: DRKS00016854).

The final dataset included 22 patients and 22 controls tested remotely, as well as 22 patients and 22 controls tested at site.

Because remote assessments became available only after the at site data collection had begun, the same patients could not be examined under both conditions. The present study therefore compares two independent, matched cohorts rather than applying a within-subject crossover design.

### Cognitive Assessment

#### CCAS-Scale

All participants were assessed using the German version A of the Cerebellar Cognitive Affective Syndrome Scale (CCAS-S). The CCAS-S consists of ten test items, including *semantic word fluency*, *phonematic word fluency*, *category switching*, *digit span forward*, *digit span backward*, *cube drawing/copying*,* similarities*,* go/no-go* and *affect*. All test items capture one or several core cognitive and affective domains typically impaired in CCAS.

In addition, the scale includes a *delayed verbal recall* item assessing episodic memory. Although episodic memory deficits are observed in some cerebellar patients, they are not considered a core feature of CCAS per se. Rather, when present, they are interpreted as indicators of additional extracerebellar (cerebral) involvement. This distinction is particularly relevant in degenerative ataxias, which frequently show extracerebellar (i.e., cerebral) pathology. Accordingly, the delayed verbal recall item serves as an indicator of cognitive deficits attributable to extracerebellar involvement [24, 33].

Each test item yields a raw score and is evaluated against a predefined pass/fail criterion. If the item-specific cut-off for the raw score is not met, the respective test item is classified as failed. Upon completion of the CCAS-S, the total number of failed test items is calculated by summing all failed items (range: 0–10). According to the authors of the original US version of the CCAS-S (Hoche et al. [24]), the number of failed items reflects the likelihood of CCAS, with one failed item indicating *possible* CCAS, two failed items indicating *probable* CCAS, and three or more failed items indicating *definite* CCAS.

In addition, the raw scores of the individual test items can be summed to obtain a total raw score (range: 0-120 points). However, the classification proposed by Hoche et al. has been shown to yield a high rate of false-positive results in healthy individuals, which can be partly attributed to effects of age and education and, to a lesser extent, sex [28, 36, 39]. To address these confounding factors, our group developed correction formulas to control for these effects [35]. These formulas predict either the expected number of failed test items *Y* (Eq. 1) or the lower bound of the prediction interval for the total raw score *Z* (Eq. 2) for an individual with a given age, level of education, and sex (see supplementary materials, M1).

#### Adaptations for Remote Administration

No major adaptations to the standard administration protocol were required, as responses to all, but one test item are given orally. For the cube drawing item, participants drew on paper and held the sheet up to their webcam. A screenshot was captured for scoring. The go/no-go item likewise required no adaptation: participants knocked on the table as instructed, which was clearly visible to the examiner via the webcam. Adaptations of the CCAS-S for teleconference-based administration have also been described by Selvadurai et al. [25]; Bolzan et al. report the same procedure for the cube drawing item, with participants drawing the cube, presenting it to the camera, and describing the remaining items as administered in the form of a structured interview without apparent differences between the two settings [26]. Some patients required assistance from a caregiver to set up the computer or install the Zoom application prior to testing.

Remote assessments were conducted using participants’ personal laptop computers with a built-in or external webcam. Headphones were used by some but not all participants. A stable internet connection was confirmed before each session, and no communication problems occurred during testing. Further details on screen size and ambient conditions were not systematically recorded.

#### Reliability of Remote CCAS-S Application

A subset of patients and healthy controls tested remotely underwent a repeated assessment using the same version of the scale to evaluate test-retest (i.e., *intra*rater; 17 patients and 19 controls) and interrater reliability (13 patients and 14 controls). Note that some participants contributed to both the test-retest and interrater reliability subsets. Consequently, the combined number of participants across these subsets may exceed the total sample size of 22 patients and 22 controls. Follow-up assessments for test-retest reliability were conducted within an interval of 18–39 days. The retest interval was deliberately varied so that potential recall effects could be examined by relating the length of the interval to the change in performance between the two sessions. For test-retest reliability, participants were assessed at both time points by the same rater. For interrater reliability, two raters assessed the CCAS-S in parallel during the same video conference – with one rater conducting the testing and rating CCAS-S performance, while the second rater only rated CCAS-S performance.

To assess test-retest (*intra*rater) and *inter*rater reliability, the intraclass correlation coefficient (ICC; absolute agreement, two-way random-effects model, average measures) with its 95% confidence interval (CI), the standard error of measurement (SEM), and the minimal detectable change at the 80% (MDC80%) and 95% (MDC95%) confidence intervals were calculated. The SEM represents the amount of error inherent in a measurement due to limitations of the instrument or variability in the measurement process, whereas the MDC indicates the smallest true change in a score that exceeds this measurement error [43, 44]. The formulas used to calculate the SEM, MDC80%, and MDC95% are provided in the supplementary material (M2).

To evaluate potential learning effects, changes (Δ) in overall test measures between baseline (T1) and retest (T2) were calculated as follows: Δ uncorrected failed test items = uncorrected failed test items at T2 minus those at T1, and Δ uncorrected total sum raw score = uncorrected total sum raw score at T2 minus that at T1. These change scores were then correlated with the time interval between T1 and T2 using Spearman’s rank correlation coefficient [45]. We hypothesized that, if learning effects were present, their magnitude would decrease with increasing intervals between T1 and T2.

Finally, in line with Hoche et al. [24], internal consistency was assessed using Cronbach’s alpha. Internal consistency reflects the degree of interrelatedness among individual test items and evaluates whether all items of the CCAS-S measure the same construct, namely CCAS. Cronbach’s alpha values were interpreted as poor (< 0.7), acceptable (≥ 0.7), good (≥ 0.8), or excellent (≥ 0.9) according to the literature [24, 46].

#### Assessment of Ataxia and Non-Ataxia Signs

For the purpose of matching the groups in terms of disease severity, two ataxia rating scales including the Scale for the Assessment and Rating of Ataxia (SARA) [47] and the International Cooperative Ataxia Rating Scale (ICARS) were assessed [41]. The SARA assessment evaluates gait, stance, sitting, speech, finger chase, the nose-finger test, rapid alternating hand movements, and the heel-shin slide. Scores range from 0 to 40, with higher scores indicating greater severity of ataxia [8]. The ICARS is comparable in structure but spans a broader scoring range (0-100 points) and, unlike the SARA, also incorporates assessment of cerebellar oculomotor dysfunction [41]. Additionally, the Inventory of Non-Ataxia Signs (INAS) [42] was applied to quantify non-ataxia symptoms. In the INAS, non-ataxic features (e.g., spasticity, sensory deficits, dystonia, hyperreflexia) are recorded and summed to generate the INAS count, where 0 indicates absence of extracerebellar signs and 16 indicates maximal extracerebellar involvement. Accordingly, the INAS count functions as a semiquantitative measure of extracerebellar symptoms [42].

#### Statistics

Histograms and results from a Shapiro-Wilk test showed that the data were not normally distributed (p-values between 0.003 and 0.567). Group comparisons of demographic data were conducted using estimation statistics (https://www.estimationstats.com). Two-sided permutation t-tests with 5000 reshuffles were calculated; the permutation t-test is robust to non-normal distributions [48]. Because the assumption of normality was not met for several outcome variables, group differences in the primary outcomes (failed test items, total sum raw score) were analyzed using rank-based 2 × 2 analyses of variance (diagnostic group × test mode), followed by Bonferroni-corrected post-hoc pairwise comparisons to control for multiple comparisons. For the ten single CCAS-S test items, p-values were corrected for multiple testing using the Bonferroni procedure within each family of comparisons. Categorical demographic and clinical variables were compared using Fisher’s exact test [35, 49]. Null hypotheses were rejected at a significance level of 0.05.

#### Use of Artificial intelligence Tools

Artificial intelligence tools (Claude, Anthropic) were used to assist with editorial revision of the manuscript and with literature search. All AI-generated suggestions were critically reviewed, validated against primary sources, and approved by the authors, who take full responsibility for the accuracy and integrity of the final manuscript.

## Results

### Demographic and Clinical Characteristics

Demographic and clinical information of matched groups is given in Table 1. Continuous variables were compared with the permutation t-test and categorical variables were compared with Fisher’s exact test. None of the comparisons was statistically significant except the comparison of educational years between patients and controls tested remotely, due to the reduced sample size, matching for educational years was limited.

#### Group Comparison with Corrected Scores

We applied the correction formulas recently published by Thieme et al. [35] (see supplementary materials, M1) to all patients and controls. The classification as *normal/abnormal* test result based on the correction formulas was then compared to the categorization according to Hoche et al. [24] in terms of sensitivity, selectivity, and rate of false positive test results.

**Fig. 1:**
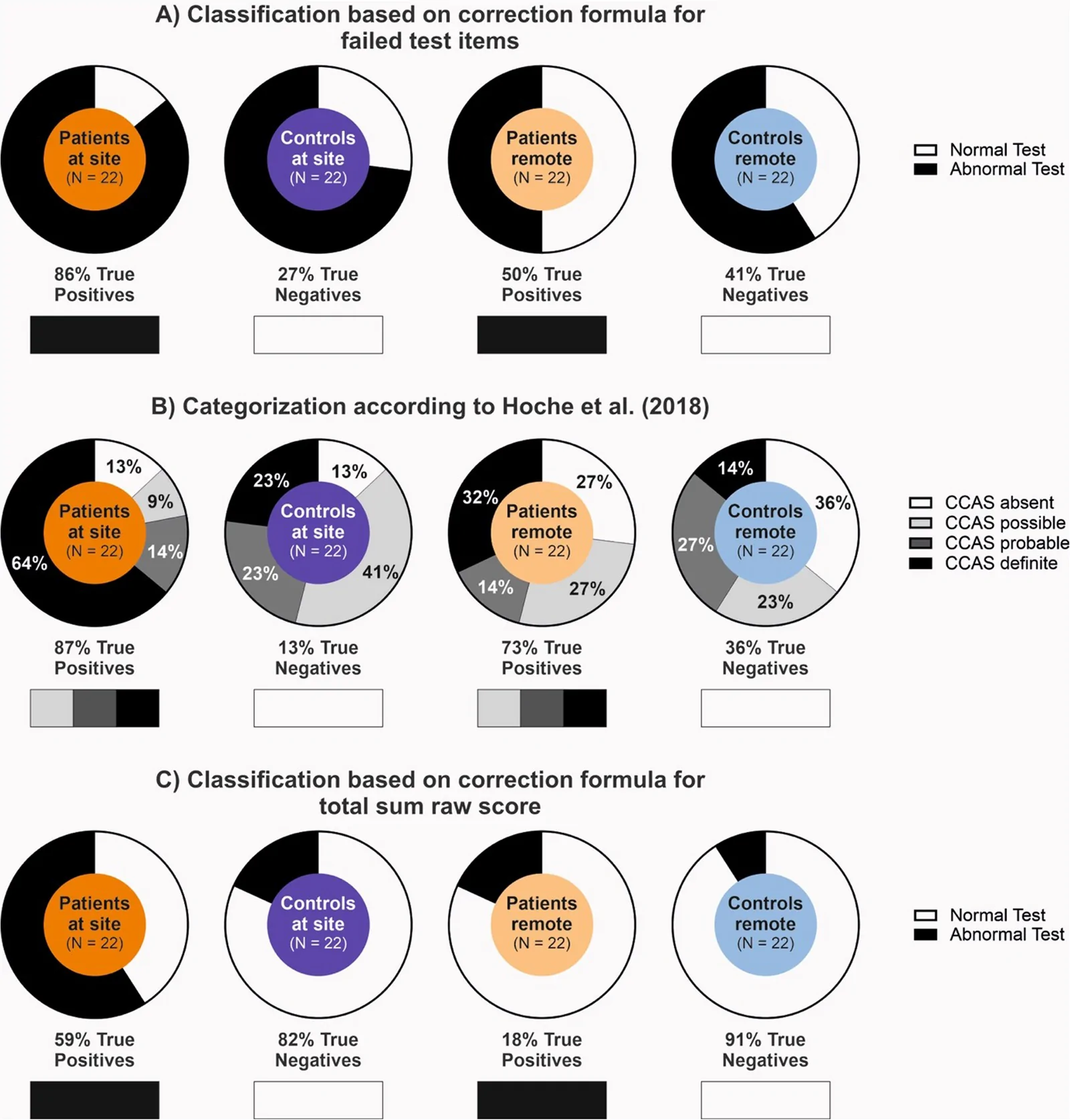
Classification as *normal/abnormal* test result based on correction formulas from Thieme et al. [35] in comparison to the original categorization as *CCAS possible/probable/definite* according to Hoche et al. (2018) [24]. (**A**) Classification based on the correction formula for failed test items: Using the correction formula, at site assessment yielded a higher proportion of true positives among patients but remote assessment yielded a higher proportion of true negatives among controls. (**B**) Categorization according to Hoche et al. [24]: Applying the CCAS categorization revealed higher true-positive rates for patients, particularly in the remote setting, whereas true-negative rates in controls were comparatively low, indicating increased misclassification, especially for controls. (**C**) Classification based on the correction formula for the total summed raw score at the 95% confidence interval level: When classification relied on the corrected total raw score, specificity was high in controls, particularly in the remote condition, while sensitivity for detecting patients was lower, most notably during remote assessment

Considering the number of failed test items, 86% of patients tested at site and 50% of patients tested remotely were considered to have an *abnormal* test when using the correction formula, corresponding to a sensitivity (i.e. the proportion of patients correctly classified as abnormal) of 86% and 50%, respectively (Fig. 1A). In contrast, according to the *uncorrected* method of Hoche et al. [24], 87% of patients tested at site and 73% of patients tested remotely were considered have an *abnormal* test to some extent (CCAS *possible/probable/definite*: patients tested at site: 9/14/64%; patients tested remotely: 27/14/32%; Fig. 1B).

Considering the number of failed test items, 73% of controls tested at site and 59% of controls tested remotely were considered to have an *abnormal* test when using the correction formula, corresponding to a selectivity (i.e. the proportion of controls correctly classified as normal) of 27% and 41%, respectively (Fig. 1A). In contrast, according to the *uncorrected* method of Hoche et al. [24], 87% of controls tested at site and 64% of controls tested remotely were considered have an *abnormal* test to some extent (CCAS *possible/probable/definite*: controls tested at site: 41/23/23%; controls tested remotely: 23/27/14%; Fig. 1B).

Using the correction formula for the total sum raw score at the 95% CI level yielded less sensitive, but more selective test results – both for the at site and the remotely tested cohort (sensitivity 59% at site and 18% remotely; selectivity 82% at site and 91% remotely; Fig. 1C).

#### Group Comparisons with Uncorrected Scores

For reliability analyses and group comparisons, uncorrected scores are reported as the primary metric. The correction formulas were developed for individual-level diagnostic classification and are therefore not intended for group-level statistical analyses. Reliability was assessed using the original test scores to evaluate the stability of the underlying measurements rather than the derived classification procedures. Next, group comparisons were carried out using the *uncorrected* scores for the number of failed test items, the total sum raw score and single test items’ raw scores. Note that groups were closely matched for demographic and clinical characteristics as described above; however, due to the reduced sample size, matching for years of education was limited.

##### Uncorrected Total Failed Test Items

**Fig. 2:**
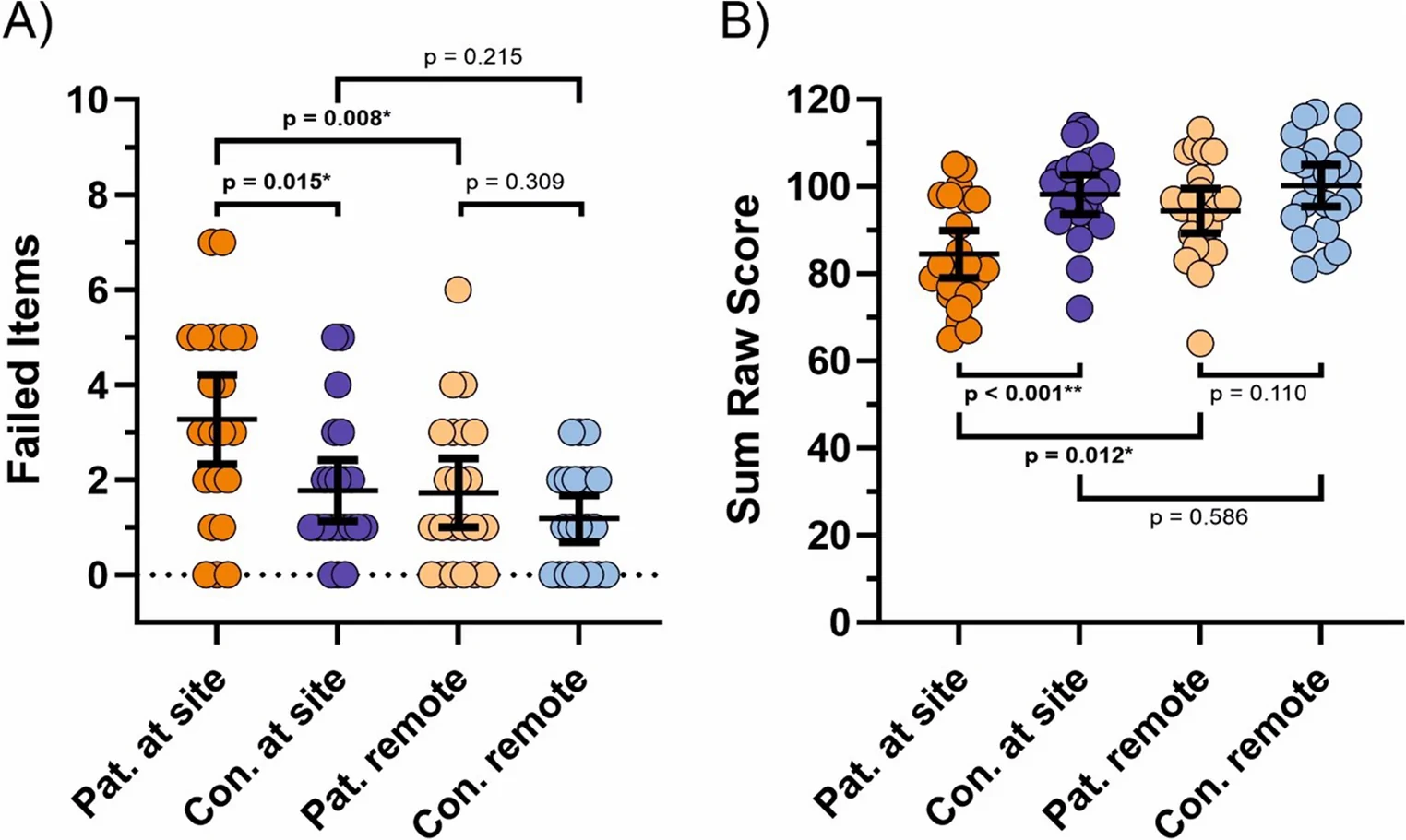
*Uncorrected* total failed test items and total sum raw score. (**A**) Patients assessed at site showed a significantly higher number of failed items compared with at site controls (*p* = .015), whereas differences between remote assessments of patients and controls were not significant. Patients tested at site also failed significantly more items than patients tested remotely (*p* = .008), while the corresponding comparison between control groups was not significant. (**B**) The total sum raw score differed significantly between patients and controls during at site assessment (*p* < .001), with a similar trend for remote assessment that did not reach statistical significance. Patients tested at site scored significantly lower than patients tested remotely (*p* = .012), whereas the two control groups did not differ significantly. All p-values refer to the Bonferroni-corrected post-hoc comparisons of the rank-based analysis of variance reported in the Results section

Patients failed on average more test items than their respective controls (patients tested at site: 3.27 ± 2.12 vs. controls tested at site: 1.77 ± 1.45; patients tested remotely: 1.73 ± 1.64 vs. controls tested remotely: 1.18 ± 1.10). A rank-based 2 × 2 analysis of variance (diagnostic group × test mode) revealed significant main effects of diagnostic group [F(1,84) = 6.119, p = .015, ηp² = 0.068] and test mode [F(1,84) = 7.792, p = .006, ηp² = 0.085], without a significant diagnostic group × test mode interaction [F(1,84) = 1.052, p = .308, ηp² = 0.012]. Bonferroni-corrected post-hoc comparisons of the rank-transformed scores (with the corresponding raw mean differences [raw MD] reported descriptively alongside) showed that patients tested at site failed significantly more test items than their respective controls [raw MD = 1.50; rank MD = 17.52, 95% CI 3.44–31.61, p = .015] and than patients tested remotely [raw MD = 1.54; rank MD = 19.11, 95% CI 5.03–33.20, p = .008], whereas the corresponding differences did not reach significance for controls tested at site versus remotely [raw MD = 0.59; rank MD = 8.84, 95% CI − 5.24–22.92, p = .215] nor for patients versus controls tested remotely [raw MD = 0.55; rank MD = 7.25, 95% CI − 6.83–21.33, p = .309] (Fig. 2A).

##### Uncorrected Total Sum Raw Score

Patients reached on average a lower total sum than their respective controls (patients tested at site: 84.55 ± 12.30 vs. controls tested at site: 98.27 ± 10.11; patients tested remotely: 94.45 ± 11.52 vs. controls tested remotely: 100.27 ± 10.79). A rank-based 2 × 2 analysis of variance revealed significant main effects of diagnostic group [F(1,84) = 13.706, p < .001, ηp² = 0.140] and test mode [F(1,84) = 4.805, p = .031, ηp² = 0.054], without a significant diagnostic group × test mode interaction [F(1,84) = 2.012, p = .160, ηp² = 0.023]. Bonferroni-corrected post-hoc comparisons showed that patients tested at site reached a significantly lower total sum raw score than their respective controls [raw MD = − 13.72; rank MD = − 25.43, 95% CI − 39.40–−11.47, p < .001] and than patients tested remotely [raw MD = − 9.90; rank MD = − 17.93, 95% CI − 31.90–−3.97, p = .012], whereas the corresponding differences did not reach significance for controls tested at site versus remotely [raw MD = − 2.00; rank MD = − 3.84, 95% CI − 17.81–10.13, p = .586] nor for patients versus controls tested remotely [raw MD = − 5.82; rank MD = − 11.34, 95% CI − 25.31–2.63, p = .110] (Fig. 2B).

##### Uncorrected Single Test Items

At the level of single test items, Bonferroni-corrected comparisons within each family of comparisons revealed significant differences between patients and controls tested at site for five of the ten test items (semantic word fluency, phonematic word fluency, category switching, similarities and affect), whereas none of the ten items differed significantly between patients and controls tested remotely. Comparing test modes within groups, no test item differed significantly between patients tested at site and patients tested remotely, while two test items (go no-go and affect) differed significantly between controls tested at site and controls tested remotely. Rank mean differences, 95% confidence intervals and Bonferroni-corrected p-values for all single test items are given in Tab. S1, and the corresponding raw score distributions are shown in Fig. S1, supplementary material.

### Test-Retest (Intrarater) and Interrater Reliability

Test-retest and interrater reliability were assessed in a subset of the remotely tested cohort only (see Methods for subgroup sizes). Because of different German CCAS-S performance of patients and controls (as shown above), test-retest (i.e. *intra*rater) and *inter*rater reliability were calculated separately for the subgroups of patients and controls. This allowed us to assess how consistently the CCAS-S measures cognitive performance in healthy and diseased participants, with the patient subgroups being of particular interest. A control group was included to check if learning effects become relevant in repeated measurements. Learning in the context of CCAS-S assessment might be impaired in patients. In the literature there is a large body of evidence for impairments in cerebellar patients for different forms of learning [50–54].

Test-retest (*intra*rater) reliability was assessed in 17 patients (range: 20–39 days; mean ± standard deviation: 28.4 ± 6.2 days) and 19 controls (18–35; 28.7 ± 6.0 days). *Inter*rater reliability was assessed on the same day in 13 patients and 14 controls, as described in the methods section. The *uncorrected* number of failed test items and the *uncorrected* total sum raw scores for test (T1) and retest [respectively the second rating assessed for interrater reliability] (T2) are displayed in Tab. S2, supplementary material.

Considering the uncorrected number of failed test items, test-retest (intrarater) reliability was good in patients (ICC: 0.87) and in controls (ICC: 0.79). The SEM (i.e. the inherent error of the G-CCAS-S) was determined to be 0.6 failed test items for patients and 0.5 for controls. This yielded a minimal detectable change at the 95% confidence interval (MDC95%) of 1.7 failed test items for patients and 1.4 for controls. Test-retest (intrarater) reliability was good considering the uncorrected total sum raw score in patients (ICC: 0.86) and in controls (0.85). These ICCs yielded a SEM of 5.1 and a MDC95% of 14.0 raw score points in patients and a SEM of 4.2 and a MDC95% of 11.6 in controls (Table 2).

**Table 2:** Test-retest (*intra*rater) and *inter*rater reliability based on uncorrected scores

Measure	Patients, tested remotely		Controls, tested remotely	
Test-retest (*intra*rater) reliability	Failed test items	Total sum raw score	Failed test items	Total sum raw score
ICC	0.87	0.86	0.79	0.85
95% CI	0.65–0.95	0.62–0.95	0.46–0.92	0.60–0.94
SEM	0.6	5.1	0.5	4.2
MDC80%	1.6	12.9	1.3	10.7
MDC95%	1.7	14.0	1.4	11.6
Measure	Patients, tested at site [35]		Controls, tested at site [35]	
Test-retest (intrarater) reliability	Failed test items	Total sum raw score	Failed test items	Total sum raw score
ICC	0.77	0.89	0.88	0.91
SEM	0.9	4.1	0.5	3.3
MDC80%	2.2	10.5	1.3	8.5
MDC95%	2.4	11.4	1.4	9.2
Measure	Patients, tested remotely		Controls, tested remotely	
Interrater reliability	Failed test items	Total sum raw score	Failed test items	Total sum raw score
ICC	0.85	0.95	0.91	0.99
95% CI	0.53–0.95	0.83–0.98	0.71–0.97	0.95-1.00
SEM	0.6	2.7	0.4	1.4
MDC80%	1.5	6.8	1.0	3.5
MDC95%	1.6	7.3	1.1	3.8
Measure	Patients, at site [35]		Controls, at site [35]	
Interrater reliability	Failed test items	Total sum raw score	Failed test items	Total sum raw score
ICC	0.88	0.95	0.59	0.86
SEM	0.8	3.4	0.9	3.6
MDC80%	2.1	8.8	2.3	9.3
MDC95%	2.2	9.5	2.5	10.1

The table shows the reliability of the remotely administered German CCAS-S obtained in the present study together with the corresponding published values for at site administration reported by Thieme et al. [35]. For test-retest (intrarater) as well as for interrater reliability, the upper block gives the values of the present remote cohort and the lower block the at site reference values. Reliability is reported for the uncorrected number of failed test items and the uncorrected total sum raw score as intraclass correlation coefficients (ICC, average measures; with 95% confidence intervals for the present study), standard error of measurement (SEM), and minimal detectable change at the 80% and 95% confidence level (MDC80%, MDC95%). Note that the reliability data of the present study are based on a subset of the remotely tested cohort only (see Results for subgroup sizes)

Abbreviations: *ICC*  intraclass correlation coefficient, *CI*  confidence interval, *SEM*  standard error of measure, *MDC80%/MDC95%* minimal detectable change at 80% or 95% confidence interval

Considering the uncorrected number of failed test items, interrater reliability was good (ICC: 0.85) in patients and excellent in controls (ICC: 0.91). The SEM was determined to be 0.6 failed test items in patients and 0.4 in controls. The MDC95% was 1.6 failed test items in patients and 1.1 in controls. Interrater reliability was excellent when considering the uncorrected total sum raw score both in patients (ICC: 0.95) and in controls (ICC: 0.99). These ICCs yielded a SEM of 2.7 in patients and 1.4 in controls and a MDC95% of 7.3 raw score points in patients and 3.8 in controls (Table 2).

Finally, possible learning effects were assessed within the group of patients and controls tested for test-retest reliability by correlating the time interval between baseline (T1) and retest (T2) with the change (Δ) of *uncorrected* failed test items or *uncorrected* total sum raw score between T1 and T2. Correlations were calculated separately for patients and controls. As retest assessments were conducted exclusively within the remotely tested cohort, these correlations reflect pooling of the respective remote subgroup only and do not include any at site tested participants. None of the correlations were statistically significant (r-values: -0.21 to 0.41; p-values: 0.09–0.98, Spearman’s rank correlation coefficient; Tab. S3, supplementary material), indicating that no substantial learning effects were present. Neither in patients nor in controls did the change in performance depend on the length of the retest interval, so that the control subgroup — included to detect recall effects that might be less pronounced in cerebellar patients — gave no indication of a differential learning effect. Given the size of the two subgroups, this analysis is sensitive only to large effects.

For comparison, Table 2 also lists the corresponding reliability coefficients for at site administration of the German CCAS-S as reported by Thieme et al. [35]. As the reliability subgroups of the present study comprised only 13 to 19 participants, the 95% confidence intervals of the present coefficients are wide (Table 2). The coefficients of the two studies are presented side by side for descriptive purposes only and were not compared statistically; the reasons are addressed in the Discussion.

## Discussion

This study evaluated remote administration of the German CCAS-S and demonstrates that remote testing is feasible. The recruited patients with cerebellar ataxia were able to participate in remote cognitive assessment with the German CCAS-S via the Zoom application.

Remote testing yielded similar results in terms of selectivity (that is the ability of the CCAS-S to identify controls correctly as controls) to those acquired in at site testing. Considering the number of failed test items, selectivity (i.e. true negatives) was 27% in controls tested at site, and somewhat higher (41%) in controls tested remotely using the correction formula for demographic influences introduced by Thieme et al. [35].

When considering the corrected total sum raw score, selectivity was improved to 82% for controls tested at site, and 91% for those tested remotely. This has also been observed in the validation trial of the German CCAS-S [35]. The current results confirm these findings and extend them to remote application of the CCAS-S.

A comparable pattern has been described for fully remote CCAS-S administration in spinocerebellar ataxias. Selvadurai et al. reported that 43% of remotely tested patients, but also 14% of remotely tested controls, met the criteria for definite CCAS. They pointed out the importance of demographic correction formulas such as those developed for the German CCAS-S as a means of improving specificity [25].

In contrast to selectivity, sensitivity differed markedly between at site and remote testing. This corresponds to the significantly lower number of failed test items and the significantly higher total sum raw score of the remotely tested patients reported above.

Considering the number of failed test items, 86% of patients tested at site and 50% of patients tested remotely were identified correctly as patients. A similar difference was observed for the total sum raw score: 59% of the at site tested patients, but only 18% of the remotely tested patients showed abnormal test results.

Notably, remotely tested patients performed better than at site tested patients despite comparable severity of motor symptoms. This suggests that the remote cohort may have been cognitively less severely affected, possibly due to selection bias. More patients having only mild cognitive dysfunction may have consented to participate in the remote testing. Another explanation for the better cognitive performance in the remotely tested cohort might be that home-based testing is less socially stressful than clinic-based testing. Neuropsychological assessments at site are conducted in artificially controlled clinical environments, which may induce elevated test anxiety — analogous to the well-known ‘white-coat effect’ in blood pressure measurement [55, 56]. Remote testing in the home environment may reduce such anxiety and thus more accurately reflect a patient’s everyday cognitive function [57]. A comparable observation has been reported for the CCAS-S in spinocerebellar ataxia type 3: Bolzan et al. found better performance in all remotely assessed subgroups than in those assessed at site, with the main effect on the total score and on four individual test items reaching statistical significance, and likewise suggested that assessment at home, in a less stressful environment and with fewer sensory distractions, may confer an advantage [26].

The reliability measures obtained in the present study provide further support for the feasibility of remote CCAS-S administration. In the remotely tested cohort, test-retest (intrarater) reliability was good in patients and in controls, both for the uncorrected number of failed test items and for the uncorrected total sum raw score, and was of similar magnitude as the values reported for at site administration in the German validation study [35] (Table 2). These findings are compatible with test-retest reliability of the CCAS-S being largely unaffected by the mode of administration. They do not, however, permit a formal statement to that effect: the two sets of estimates derive from independent samples that differ in cohort composition, and the reliability subgroups of the present study were small, so that the corresponding confidence intervals are wide. A different pattern emerged for interrater reliability. In the remotely tested cohort, interrater reliability was good for the uncorrected number of failed test items in patients and excellent in controls, and excellent for the uncorrected total sum raw score in both groups. The values obtained in patients correspond closely to those reported for at site administration, whereas in controls the remote values were higher (Table 2). This difference is unlikely to reflect the mode of administration. It is more plausibly explained by a difference in the interrater design of the two studies: Thieme et al. assessed the same participants on two separate days by two different raters, so that their estimate most likely also contains the within-participant variability between the two test occasions, whereas in the present study two raters scored the same test session simultaneously. The latter design removes this source of variability and therefore most likely yields higher agreement. Because the two coefficients thus refer to different sources of error, and because the reliability subgroups comprised only 13 to 23 participants in either study, the reliability coefficients of the two studies were not compared statistically. The present data showed that the correction formulas also improve selectivity when the scale is administered remotely. The question of measurement equivalence between modalities is of immediate practical relevance, as studies have begun to combine at site and virtual CCAS-S assessments within the same cohort [26]; if data from both settings are pooled, systematic differences between modalities could bias group comparisons.

Despite these findings, the present study has several limitations. The first concerns the already mentioned potential selection bias of patients in the remotely tested cohort. Moreover, although the proportion of cerebellar-pure and cerebellar-plus diagnoses was matched between the two patient cohorts, the composition within the cerebellar-plus group differed: the at site tested cohort included more patients with SCA3, SCA13 and SCA48, which are frequently associated with more pronounced cognitive involvement [58]. This may have contributed to the observation that at site tested patients failed more items, especially delayed verbal recall, which serves as an indicator of cognitive deficits attributable to extracerebellar involvement. Thus, stronger deficits in the at site tested cohort may reflect underlying disease characteristics rather than differences in test modality. Future studies should confirm and extend the current findings by including more severely cognitively affected patients in remote testing with CCAS-S. Also, more uniform cohorts in terms of molecular diagnoses could help to evaluate the diagnostic abilities of the CCAS-S more precise. Another limitation is the comparably small sample size. In the current study, each group contained 22 participants limiting statistical power. For comparison: The German validation study contained over 200 patients and over 200 controls [35]. Other studies testing the CCAS-S had a similar sample size [28, 29, 37]. In summary, the current data support the general feasibility and robustness of remote CCAS-S administration. Remote CCAS-S administration showed good test-retest and excellent interrater reliability in the present sample and may therefore be suitable for low-threshold repeated cognitive evaluations in ataxic patients. However, the present findings suggest that remote and at site CCAS-S data may not be directly comparable and therefore should be pooled with caution. 

## Supplementary Information

Below is the link to the electronic supplementary material.

Supplementary Material 1 (DOCX 291 KB)

Supplementary Material 2 (DOCX 285 KB)

## Data Availability

All data supporting the findings of this study are available within the paper and its Supplementary Information.

## Declarations

### Study Registration

The study has prospectively been registered at the German Clinical Study Register (https://www.drks.de; DRKS-ID: DRKS00016854).
