# Supplementary material for: Remote Online-Based Application of the Cerebellar Cognitive Affective Syndrome Scale
Source: Cerebellum. 2026 Oct 7;25(5):152. doi: 10.1007/s12311-026-02097-w (PMC13645978; doi:10.1007/s12311-026-02097-w)
Supplement: Supplementary file 1 [file 12311_2026_2097_MOESM1_ESM.docx]

**Supplementary materials to Remote online-based application of the Cerebellar Cognitive Affective Syndrome Scale**

**Sönke Konrad^1,2,^, Johanna Aschmann^,2^,** **Raquel van der Veen^1,2,^ Kerstin Rubarth^3^, Frank Konietschke^3,4^, Dagmar Timmann^1,2^, Andreas Thieme^1,2,5^**

1) Department of Neurology, Essen University Hospital, University of Duisburg-Essen, Essen, Germany
2) Center for Translational Neuro- and Behavioral Sciences (C-TNBS), Essen University Hospital, University of Duisburg-Essen, Essen, Germany
3) Institute of Biometry and Clinical Epidemiology, Charité - University Medicine Berlin, Corporate Member of Freie University, Berlin, Germany
4) Berlin Institute of Health (BIH), Berlin, Germany
5) Department of Neurology, Knappschaft Kliniken Bottrop, Bottrop, Germany

**M1. Correction formulas for the number of failed items and total sum raw score**

**from Thieme et al. 2025 [1]**

*Predicted failed test items:*

*Equation 1:*	Y = e ^(0.99 - 0.13 * sex + 0.01 * age - 0.1 * edu)^

*Predicted lower bound of total sum raw score:*

*Equation 2:*	Z = 93.3 + 2.92 * sex – 0.12 * age + 0.76 * edu – z_1-α/2_ * 7.93 * $\sqrt{1.18-0.02*edu}$

where *sex* is 0 if participant is a male, and *sex* is 1 if participant is a female; *age* is the age of the participant in years; *edu* is the educational years of the participant.

If a subject shows a number of failed test items exceeding the predicted value *Y*, or a total sum score below the lower bound *Z* of the prediction interval for the total sum score, the test result is classified as abnormal, i.e. pathological (for details, see Thieme et al. 2025 [1]).

**M2. Formulas for calculation of the standard error of measure and the minimal detectable change at the 80% and 95% confidence interval.**

Standard error of measure

SEM = SD_pooled_ * $\sqrt{1-ICC}$

*where SD_pooled_ is the arithmetic mean of the standard deviations of the 1^st^ and 2^nd^ test session*

Minimal detectable change at the 80% confidence interval

MDC80% = 1.28 * MDC *$\surd2$

*where MDC = SEM ** $\surd2$

Minimal detectable change at the 80% confidence interval

MDC95% = 1.96 * MDC *$\surd2$

*where MDC = SEM ** $\surd2$

**Figure S1**

**Figure S1: uncorrected raw scores of remote and at site CCAS testing**

Figure S1 shows the uncorrected raw scores of CCAS testing in the different cohorts, comparing patients tested remote to controls tested remoted, phonematic fluency and digit span backwards showed statistically significant differences, comparing patients tested remote to those at site, category switching and verbal recall showed statistically significant differences and when comparing patients tested at site to controls tested at site, semantic and phonematic fluency, category switching, similarities and affect showed statistically significant differences.

**Tab. S7. Unpaired mean differences and results of two-sided permutation t-tests.**

| **Test item** | **Patients tested remote vs.**  **Controls tested remote**  UMD, 95% CI  p-value | **Patients tested at site vs.**  **Controls tested at site**  UMD, 95% CI  p-value | **Patients tested remote vs.**  **Patients tested at site**  UMD, 95% CI  p-value | **Controls tested remote vs.**  **Controls tested at site**  UMD, 95% CI  p-value |
| --- | --- | --- | --- | --- |
| **Semantic fluency** | 1.7; 0.9, 4.14  0.2 | **4.2; 1.9, 6.6**  **0.001** | -2.3; 4.8, 0.4  0.09 | 0.2; -1.8, 2.5  0.8 |
| **Phonematic fluency** | **3.1; 0.7, 5.4**  **0.017** | **4.2; 2.3, 6.3**  **0.0004** | -2.0; -4.0, 0.0  0.07 | -0.8; -3.0, 1.6  0.5 |
| **Category switching** | 0.7; 0.8, 2.0  0.3 | **2.7; 0.9, 4.4**  **0.003** | **-2.1; 3.7, -0.5**  **0.02** | -1.0; -2.6, 1.0  0.3 |
| **Digit span forward** | -0.2; -0.8, 0.3  0.4 | -0.5; -1.2; 0.2  0.2 | -0.4; -1.1, 0.1  0.1 | -0.7; -1.4, -0.1  0.03 |
| **Digit span backward** | **0.7; 0.09, 1.18**  **0.018** | 0.1; -0.5, 0.6  0.6 | 0.2; -0.3, 0.7  0.4 | -0.4; -1.0, 0.2  0.2 |
| **Cube drawing** | 0.7; -0.7, 2.0  0.3 | 0.2; -1.3, 1.6  0.8 | -0.3; -1.9, 1.1  0.6 | -0.8; -2.2, 0.5  0.2 |
| **Verbal recall** | -1.4; -3.2, -0.1  0.07 | 0.6; -0.7, 1.8  0.3 | -1.7; -2.9, -0.5  0.008 | 0.3; -1.1, 2.0  0.7 |
| **Similarities** | 0.4; -0.05, 0.9  0.06 | **0.9; 0.5, 1.3**  **0.0** | -0.4; -1.0, 0.1  0.1 | 0.09; -0.2, 0.4  0.4 |
| **Go/no-go** | 0.2; -0.2, 0.5  0.2 | -0.3; -0.8, 0.1  0.1 | -0.3; -0.7, 0.1  0.1 | **-0.8; -1.2, -0.5**  **0.0004** |
| **Affect** | -0.2, -1.0, 0.5  0.5 | **1.6; 1.1, 2.4**  **0.0** | -0.6; -1.5, 0.1  0.1 | **1.2; 0.7, 1.7**  **0.0** |
| **Overall Scores** | **Patients tested remote vs.**  **Controls tested remote**  UMD, 95% CI  p-value | **Patients tested remote vs.**  **Patients tested at site**  UMD, 95% CI  p-value | **Patients tested at site vs.**  **Patients tested at site**  UMD, 95% CI  p-value | **Controls tested remote vs.**  **Controls tested at site**  UMD, 95% CI  p-value |
| **Failed test items** | 0.5; -1.5, 0.2 | **13.7; 7.1, 20.2**  **0.0** | **1.6; 0.4, 2.6**  **0.008** | -0.6, -1.4, 0.09  0.1 |
| **Total Sum Score** | 5.8; -0,5, 12,3  0,08 | **-1.5; -2.6, -0.5**  **0.007** | **-9.9, 16.2, -2.5**  **0.008** | -2.0; -8.1, 4.0  0.5 |

The UMD is the unpaired mean difference, and 95% CI ist the interval in which 95% of results lie within. If differences were significant they are marked in bold.

**Tab. S8. Total sum raw score and failed test items for test and retest.**

| **Test-retest reliability** | | | |
| --- | --- | --- | --- |
| **Failed test items** | | **Total sum raw score** | |
| **T1** | **T2** | **T1** | **T2** |
| 1.5 ± 1.7 | 1.7 ± 1.7 | 96.7 ± 13.5 | 98.7 ± 14.5 |
| **Interrater reliability** | | | |
| **Failed test items** | | **Total sum raw score** | |
| **T1** | **T2** | **T1** | **T2** |
| 1.7 ± 1.3 | 2.0 ± 1.7 | 96.0 ± 9.6 | 95.6 ± 11.4 |

The Table shows the means ± standard deviations the total sum raw score and the number of failed test items for test and retest respectively for the first and second rating and the number of failed test items for interrater reliability for the first and second rating.

**Tab. S9. Examination of possible learning effects in the test-retest cohort.**

|  | **Patients** | **Controls** |
| --- | --- | --- |
| **Failed test items** | R = -0.07  p = 0.79 | R = 0.41  p = 0.09 |
| **Total sum raw score** | R = -0.01  p = 0.98 | R = -0.21  p = 0.39 |

The Table shows spearman’s rank correlation coefficient for each group and the corresponding p-value.

**References**

1.	Thieme, A., et al., *Optimizing selectivity of the Cerebellar Cognitive Affective Syndrome Scale by use of correction formulas, and validation of its German version.* J Neurol, 2025. **272**(5): p. 343.
