# Supplementary material for: Remote Online-Based Application of the Cerebellar Cognitive Affective Syndrome Scale
Source: Cerebellum. 2026 Oct 7;25(5):152. doi: 10.1007/s12311-026-02097-w (PMC13645978; doi:10.1007/s12311-026-02097-w)
Supplement: Supplementary file 2 [file 12311_2026_2097_MOESM2_ESM.docx]

**Supplementary materials to**

**Remote online-based application of the Cerebellar Cognitive Affective Syndrome Scale**

**Sönke Konrad^1,2^, Johanna Aschmann^1,2^,** **Raquel van der Veen^1,2,^ Kerstin Rubarth^3^, Frank Konietschke^3,4^, Dagmar Timmann^1,2^, Andreas Thieme^1,2,5^**

1) Department of Neurology, Essen University Hospital, University of Duisburg-Essen, Essen, Germany
2) Center for Translational Neuro- and Behavioral Sciences (C-TNBS), Essen University Hospital, University of Duisburg-Essen, Essen, Germany
3) Institute of Biometry and Clinical Epidemiology, Charité - University Medicine Berlin, Corporate Member of Freie Universität Berlin and Humboldt Universität zu Berlin, Berlin, Germany
4) Berlin Institute of Health (BIH), Berlin, Germany
5) Department of Neurology, Knappschaft-Kliniken Bottrop, Academic Teaching Hospital of the University of Duisburg-Essen, Bottrop, Germany

**M1. Correction formulas for the number of failed items and total sum raw score**

**from Thieme et al. 2025 [1]**

*Predicted failed test items:*

*Equation 1:*	Y = e ^(0.99 - 0.13 * sex + 0.01 * age - 0.1 * edu)^

*Predicted lower bound of total sum raw score:*

*Equation 2:*	Z = 93.3 + 2.92 * sex – 0.12 * age + 0.76 * edu – z_1-α/2_ * 7.93 * $\sqrt{1.18-0.02*edu}$

where *sex* is 0 if participant is a male, and *sex* is 1 if participant is a female; *age* is the age of the participant in years; *edu* is the educational years of the participant.

If a subject shows a number of failed test items exceeding the predicted value *Y*, or a total sum score below the lower bound *Z* of the prediction interval for the total sum score, the test result is classified as abnormal, i.e. pathological (for details, see Thieme et al. 2025 [1]).

**M2. Formulas for calculation of the standard error of measure and the minimal detectable change at the 80% and 95% confidence interval.**

Standard error of measure

SEM = SD_pooled_ * $\sqrt{1-ICC}$

*where SD_pooled_ is the arithmetic mean of the standard deviations of the 1^st^ and 2^nd^ test session/rating*

Minimal detectable change at the 80% confidence interval

MDC80% = 1.28 * MDC *$\surd2$

*where MDC = SEM ** $\surd2$

Minimal detectable change at the 95% confidence interval

MDC95% = 1.96 * MDC *$\surd2$

*where MDC = SEM ** $\surd2$

**Figure S1**

**Figure S1: Uncorrected raw scores of the single CCAS-S test items in remote and at site testing.**

Figure S1 shows the uncorrected raw scores of the ten single CCAS-S test items in the four cohorts. Group differences were analysed with rank-based analyses of variance; the p-values reported here are Bonferroni-corrected across the ten test items within each family of comparisons and are given in full in Tab. S1. Comparing patients and controls tested at site, semantic word fluency, phonematic word fluency, category switching, similarities and affect differed significantly. Comparing patients and controls tested remotely, none of the ten test items differed significantly. Comparing test modes within groups, no test item differed significantly between patients tested at site and patients tested remotely, whereas go no-go and affect differed significantly between controls tested at site and controls tested remotely.

**Tab. S1. Rank mean differences and results of rank-based ANOVA (Bonferroni-corrected across the 10 test items within each comparison).**

| **Test item** | **Patients tested remotely vs.**  **Controls tested remotely**  Rank MD, 95% CI  p-value | **Patients tested at site vs.**  **Controls tested at site**  Rank MD, 95% CI  p-value | **Patients tested remotely vs.**  **Patients tested at site**  Rank MD, 95% CI  p-value | **Controls tested remotely vs.**  **Controls tested at site**  Rank MD, 95% CI  p-value |
| --- | --- | --- | --- | --- |
| **Semantic fluency** | -10.32; -24.32, 3.68  1.000 | **-22.91; -36.91, -8.91**  **0.020** | 12.79; -1.20, 26.79  0.730 | 0.20; -13.79, 14.20  1.000 |
| **Phonematic fluency** | -16.14; -29.98, -2.29  0.230 | **-26.05; -39.89, -12.20**  **<0.001** | 12.64; -1.21, 26.48  0.730 | 2.73; -11.12, 16.57  1.000 |
| **Category switching** | -7.29; -21.51, 6.92  1.000 | **-22.66; -36.88, -8.44**  **0.020** | 16.02; 1.80, 30.24  0.280 | 0.66; -13.56, 14.88  1.000 |
| **Digit span forward** | 3.41; -11.01, 17.82  1.000 | 9.68; -4.73, 24.09  1.000 | 8.18; -6.23, 22.59  1.000 | 14.46; 0.04, 28.87  0.490 |
| **Digit span backward** | -15.91; -30.05, -1.76  0.280 | -1.64; -15.78, 12.51  1.000 | -4.50; -18.64, 9.64  1.000 | 9.77; -4.37, 23.92  1.000 |
| **Cube drawing** | -6.52; -20.95, 7.91  1.000 | -1.61; -16.04, 12.82  1.000 | 3.48; -10.95, 17.91  1.000 | 8.39; -6.04, 22.82  1.000 |
| **Verbal recall** | 12.73; -1.74, 27.19  0.840 | -4.27; -18.74, 10.19  1.000 | 20.50; 6.04, 34.96  0.060 | 3.50; -10.96, 17.96  1.000 |
| **Similarities** | -9.57; -22.09, 2.95  1.000 | **-23.02; -35.55, -10.50**  **<0.001** | 11.02; -1.50, 23.55  0.840 | -2.43; -14.95, 10.09  1.000 |
| **Go/no-go** | -5.93; -19.12, 7.26  1.000 | 9.21; -3.99, 22.40  1.000 | 9.73; -3.46, 22.92  1.000 | **24.86; 11.67, 38.06**  **<0.001** |
| **Affect** | 5.36; -7.15, 17.88  1.000 | **-31.91; -44.42, -19.40**  **<0.001** | 10.36; -2.15, 22.88  1.000 | **-26.91; -39.42, -14.40**  **<0.001** |
| **Overall Scores** | **Patients tested remotely vs.**  **Controls tested remotely**  **Rank MD, 95% CI**  **p-value** | **Patients tested at site vs.**  **Controls tested at site**  **Rank MD, 95% CI**  **p-value** | **Patients tested remotely vs.**  **Patients tested at site**  **Rank MD, 95% CI**  **p-value** | **Controls tested remotely vs.**  **Controls tested at site**  **Rank MD, 95% CI**  **p-value** |
| **Failed test items** | 7.25; -6.83, 21.33  0.309 | **17.52; 3.44, 31.61**  **0.015** | **-19.11; -33.20, -5.03**  **0.008** | -8.84; -22.92, 5.24  0.215 |
| **Total Sum Score** | -11.34; -25.31, 2.63  0.110 | **-25.43; -39.40, -11.46**  **<0.001** | **17.93; 3.96, 31.90**  **0.012** | 3.84; -10.13, 17.81  0.586 |

Rank MD is the rank mean difference from the rank-based ANOVA (diagnostic group x test mode); the corresponding p-values are Bonferroni-corrected across the test items within each comparison (10 items for the single test item comparisons above; not applicable for the two Overall Scores below) and are marked in bold if significant (p < .05).

**Tab. S2. Total sum raw score and failed test items for test and retest.**

| **Test-retest reliability** | | | |
| --- | --- | --- | --- |
| **Failed test items** | | **Total sum raw score** | |
| **T1** | **T2** | **T1** | **T2** |
| 1.5 ± 1.7 | 1.7 ± 1.7 | 96.7 ± 13.5 | 98.7 ± 14.5 |
| **Interrater reliability** | | | |
| **Failed test items** | | **Total sum raw score** | |
| **T1** | **T2** | **T1** | **T2** |
| 1.7 ± 1.3 | 2.0 ± 1.7 | 96.0 ± 9.6 | 95.6 ± 11.4 |

The Table shows the means ± standard deviations of the total sum raw score and the number of failed test items for test and retest respectively and the number of failed test items for interrater reliability for test and retest respectively.

**Tab. S3. Examination of possible learning effects in the test-retest cohort.**

|  | **Patients** | **Controls** |
| --- | --- | --- |
| **Failed test items** | R = -0.07  p = 0.79 | R = 0.41  p = 0.09 |
| **Total sum raw score** | R = -0.01  p = 0.98 | R = -0.21  p = 0.39 |

The Table shows spearman’s rank correlation coefficient for each group and the corresponding p-value.

**References**

1.	Thieme, A., et al., *Optimizing selectivity of the Cerebellar Cognitive Affective Syndrome Scale by use of correction formulas, and validation of its German version.* J Neurol, 2025. **272**(5): p. 343.
